# Iron-sparing Response of *Mycobacterium avium *subsp. *paratuberculosis *is strain dependent

**DOI:** 10.1186/1471-2180-10-268

**Published:** 2010-10-22

**Authors:** Harish K Janagama, Senthilkumar, John P Bannantine, Abirami Kugadas, Pratik Jagtap, LeeAnn Higgins, Bruce A Witthuhn, Srinand Sreevatsan

**Affiliations:** 1 Department of Veterinary Population Medicine, University of Minnesota, Saint Paul MN, USA; 2 Department of Veterinary and Biomedical Sciences, University of Minnesota, Saint Paul MN, USA; 3 National Animal Disease Center, Agricultural Research Service, United States Department of Agriculture, Ames, IA, USA; 4 Minnesota Supercomputing Institute for Advanced Computational Research, University of Minnesota, Minneapolis MN, USA; 5 Department of Biochemistry, Molecular Biology and Biophysics, University of Minnesota, Minneapolis MN, USA; 6 Department of Animal Biotechnology, Madras Veterinary College, Chennai, India

## Abstract

**Background:**

Two genotypically and microbiologically distinct strains of *Mycobacterium avium *subsp. *paratuberculosis *(MAP) exist - S and C MAP strains that primarily infect sheep and cattle, respectively. Concentration of iron in the cultivation medium has been suggested as one contributing factor for the observed microbiologic differences. We recently demonstrated that S strains have defective iron storage systems, leading us to propose that these strains might experience iron toxicity when excess iron is provided in the medium. To test this hypothesis, we carried out transcriptional and proteomic profiling of these MAP strains under iron-replete or -deplete conditions.

**Results:**

We first complemented *M. smegmatis*Δ*ideR *with IdeR of C MAP or that derived from S MAP and compared their transcription profiles using *M. smegmatis mc*^*2*^*155 *microarrays. In the presence of iron, sIdeR repressed expression of *bfrA *and MAP2073c, a ferritin domain containing protein suggesting that transcriptional control of iron storage may be defective in S strain. We next performed transcriptional and proteomic profiling of the two strain types of MAP under iron-deplete and -replete conditions. Under iron-replete conditions, C strain upregulated iron storage (BfrA), virulence associated (Esx-5 and antigen85 complex), and ribosomal proteins. In striking contrast, S strain downregulated these proteins under iron-replete conditions. iTRAQ (isobaric tag for relative and absolute quantitation) based protein quantitation resulted in the identification of four unannotated proteins. Two of these were upregulated by a C MAP strain in response to iron supplementation. The iron-sparing response to iron limitation was unique to the C strain as evidenced by repression of non-essential iron utilization enzymes (aconitase and succinate dehydrogenase) and upregulation of proteins of essential function (iron transport, [Fe-S] cluster biogenesis and cell division).

**Conclusions:**

Taken together, our study revealed that C and S strains of MAP utilize divergent metabolic pathways to accommodate in vitro iron stress. The knowledge of the metabolic pathways these divergent responses play a role in are important to 1) advance our ability to culture the two different strains of MAP efficiently, 2) aid in diagnosis and control of Johne's disease, and 3) advance our understanding of MAP virulence.

## Background

*Mycobacterium avium *subsp. *paratuberculosis *(MAP), the causative agent of Johne's disease (JD) of ruminants, often requires eight to sixteen weeks to see colonies in culture - a major hurdle in the diagnosis and therefore in implementation of optimal control measures. Unlike other mycobacteria, which mobilize iron via mycobactins, MAP is unable to produce detectable mycobactin in vitro or in vivo [1-3]. Although the reasons for the in vitro mycobactin dependency of MAP are currently unknown, we have recently shown that the mycobactin (*mbt*) operon promoter is active and that the mycobactin genes are transcribed by MAP inside macrophages [4] and in tissues of naturally infected animals (accepted for publication in BMC Genomics).

Pathogenic mycobacteria encounter a wide variety of stressors inside the host cells and their ability to overcome iron deprivation and iron toxicity represents a major virulence determinant [5]. Transcript and protein profiling of MTB and other pathogens in response to in vitro iron stress is well documented [6-9]. While MAP transcriptome or proteome profiles in response to heat shock, pH, oxidative stress, hypoxia, and nutrient starvation have been demonstrated [10-12], stress responses to iron supplementation or starvation are lacking.

Iron dependent regulator (IdeR) has been very well studied as a global regulator involved in maintaining iron homeostasis in *Mycobacterium tuberculosis *(MTB) [13]. Recently we have demonstrated that IdeR of MAP in the presence of iron recognizes a consensus sequence on the promoter called "iron box" and regulates expression of genes involved in iron acquisition (*mbt*) and storage (*bfrA*). More interestingly, we demonstrated that polymorphisms in the promoter of iron storage gene (*bfrA*) in S MAP strains relative to C MAP strains results in a differential gene regulation [4]. IdeR dependent repression of *bfrA *in the presence of iron suggests variations in iron storage mechanisms and/or iron requirements in cattle and sheep MAP strains.

Comparative genomic hybridizations, short sequence repeat analysis and single nucleotide polymorphisms of MAP isolates obtained from diverse host species have established and indexed genomic differences between C and S strains of MAP [14-19]. Phylogenetic analysis of sequences has identified C and S strains as separate pathogenic clones that share a common ancestor [20-23]. Furthermore, cellular infection studies show distinctive phenotypes between the two MAP strain types [24,25]. We also recently demonstrated that S strains have defective iron storage systems, leading us to propose that these strains might experience iron toxicity when excess iron is provided in the medium [4]. Taken together, the literature suggests that MAP strains vary in their iron dependent gene regulation. To test this further, we profiled their transcriptomes and proteomes in response to iron and demonstrated that iron induced metabolic pathways are significantly diverse.

## Methods

### Bacterial strains, DNA manipulations and media

*Mycobacterium avium *subsp. *paratuberculosis *strains MAP1018 (C MAP) and MAP7565 (S MAP) were grown in Middlebrook 7H9 supplemented with OADC enrichment medium and mycobactin J (2 mg/mL; Allied Monitor, Fayette, MO).

To test the hypothesis that gene regulation may be dependent on iron availability MAP strains were grown in Middlebrook 7H9 medium without mycobactin J or Sauton medium (0.5 g KH_2_PO_4_, 0.5 g MgSO_4_, 4.0 g L-asparagine, 60 ml glycerol, 0.05 g ferric ammonium citrate, 2.0 g citric acid, 0.1 ml 1% (w/v) ZnSO_4 _and 2.5 ml 20% Tween 80 in 1 liter). Growth of MAP strains in the absence of mycobactin J took over 6 months to provide sufficient material for proteomics and transcriptional profiling. For iron restriction, 2,2'-dipyridyl (Sigma Aldrich, St. Louis, MO) was added at a concentration of 200 μM.

MAP7565 and MAP1018 have been genotyped by SSR as well as comparative genomics using oligoarrays. They represent the typical genomotypes of sheep and cattle strains, respectively [18] and show distinct phenotypes in both human and bovine macrophages [24,25].

*M. smegmatis *(mc^2^155) and *E. coli *TOP10F (Invitrogen Corporation, Carlsbad, CA) competent cells were grown in Luria Bertani (LB) medium and antibiotics (kanamycin (20 μg/ml) or hygromycin (100 μg/ml)) were added when necessary. The open reading frames of *ideR *(MAP2827) derived from C or S MAP strains were cloned into pSM417 and *M. smegmatis*Δ*ideR *(SM3) was complemented as previously reported [4]. Briefly, MAP2827 from MAP1018 (c*ideR*) or MAP 7565 (s*ideR*) was amplified via PCR using primers that carried restriction sites for *BamHI *and *HindIII*. Amplified products were double digested with *BamHI *and *HindIII *and ligated into a pre digested (*BamHI *and *HindIII*) expression plasmid pSM417. Accuracy of the ligation and orientation of MAP2827 in pSM417 was verified by sequencing. SM3 was transformed with pSM417 carrying MAP2827 from C or S MAP strains.

A seed stock from logarithmically grown (OD_600 _= 1.0) cultures were diluted to fresh medium to yield an OD_600 _= 0.1. These were grown in various aliquots under constant shaking (120 rpm) at 37°C. These cultures were monitored for their growth at weekly intervals by measuring their absorbance at 600 nm wave length using SpectraMax M2 (Molecular Devices, Sunnyvale, CA) until they reached an absorbance of 1.0 (Additional file 1, Figure S1). At this point, the cultures were then pelleted, washed in ice cold 1XPBS and re-suspended in fresh culture medium (with or without the addition of 2,2'-dipyridyl (Sigma Aldrich, St. Louis, MO)). Dipyridyl was added at a concentration of 200 μM. Following three hours of incubation at 37°C under constant shaking, cells were pelleted and washed with ice cold 1X PBS and either used in microarrays or iTRAQ. The detailed experimental design is provided as Additional file 1, Figure S2.

### Nucleic acid and protein extraction

Log phase MAP or *M. smegmatis *cultures were pelleted, washed and re-suspended in fresh culture medium with or without 200 μM of 2,2'-dipyridyl. The cultures were incubated at 37°C with shaking for 3 hr. immediately prior to RNA and protein extraction.

For RNA, cells were homogenized in Mini bead-beater for 4 min. by adding 0.3 ml of 0.1 mm sterile RNase-free zirconium beads followed by extraction using Trizol (Invitrogen, Carlsbad, CA). All samples were treated with RNase-free DNase I (Ambion, Inc., Austin, TX) to eliminate genomic DNA contamination. The purity and yield of total RNA samples was confirmed using Agilent 2100E Bioanalyzer (Agilent Technologies, Inc., Santa Clara, CA). RNA was stored at -80 until used in microarrays and real time RT-PCR assays.

For protein, cells were re-suspended in minimal quantity (250 μL) of iTRAQ dissolution buffer (0.5 M TEAB pH 8.5) and 0.1% SDS. The solution was transferred to a 2 ml screw cap tube containing 0.1 mm zirconium beads (Biospec) and disrupted in minibead beater (Biospec) for 4 × 1 minute pulses with samples kept on ice between pulses. The lysate was then centrifuged at 12,000 × g for 10 minutes at 4°C. Supernatant was transferred to a fresh tube without disturbing the pellet and used in iTRAQ labeling for detection of proteome (Additional file 1, Figure S3).

### Microarray experiments

Gene expression profiling of S (1018) and C (7565) MAP strains was performed using MAP K-10 microarrays obtained from Dr. Michael Paustian, NADC, IA. Expression profiling of *M. smegmatis*Δ*ideR *complemented with c or s*ideR *was carried out using *M. smegmatis mc*^*2*^*155 *arrays provided via Pathogen Functional Genomics Resource Center (PFGRC) at J. Craig Venter Institute (JCVI). Array hybridizations and analyses were performed as described previously and according to the protocols established at PFGRC with minor modifications [26] and according to MIAME 2.0 guidelines.

Briefly, synthesis of fluorescently labeled cDNA (Cyanine-3 or Cyanine-5) from total RNA and hybridizations of labeled cDNA to MAP K-10 or *mc*^*2*^*155 *oligoarray was performed. Microarray hybridizations were performed from cDNA isolated from two independent experiments. On each independent occasion, bacterial cultures growing under iron-replete or iron-limiting medium were used for RNA extractions, cDNA labeling and array hybridizations. Each slide was competitively hybridized with cDNA obtained from iron-replete (labeled with cy3 or cy5) and iron-limiting growth medium (counter labeled with cy5 or cy3) to reveal relative expressional differences. About 4 μg (2 μg each from iron limitation or sufficient) of cDNA was used to hybridize onto the array. However, if the cDNA yield is low for a sample the RNA from the same sample was used to synthesize more cDNA, pooled and labeled onto the arrays. Hybridized slides were scanned using HP Scan array 5000 (PerkinElmer Inc., Waltham, MA). The images were processed and numerical data was extracted using the microarray image analysis software, BlueFuse (BlueGnome Ltd, Cambridge) and TM4 microarray suite available through JCVI. Genes differentially regulated at a fold change of 1.5 or greater were identified at a false discovery rate of 1% by Statistical Analysis of Microarrays (SAM) program [26]. Genes that showed a fold change 1.5 or greater in all the replicate arrays were retained and reported as being up- or downregulated in the presence of iron.

### Realtime RT-PCR

RNA isolated from MAP strains grown under iron-replete or iron-limiting growth medium was used in real time RT-PCR assays. Genes were selected based on their diverse roles and microarray expression pattern. Selected genes included siderophore transport (MAP2413c, MAP2414c), *esx-3 *secretion system (MAP3783, MAP3784), aconitase (MAP1201c), fatty acid metabolism (MAP0150c) and virulence (MAP0216, MAP3531c, MAP1122 and MAP0475). RNA was treated with DNaseI (Ambion, Austin, TX) and one step Q-RT PCR was performed using QuantiFast SYBR Green mix (Qiagen, Valencia, CA) and gene specific primers (Additional file 1, Table S1) in a Lightcycler 480 (Roche, Indianapolis, IN).

### iTRAQ experiments

Protein extracted from the two MAP strains grown in iron-replete or iron-limiting medium was used in iTRAQ analysis (Additional file 1, Figure S3). iTRAQ labeling and protein identification was carried out as described previously with minor modifications [27].

Briefly, cell lysate was quantified using the bicinchoninic acid (BCA) protein assay (Pierce, Rockford, IL) prior to trypsin digestion. Peptides were labeled with iTRAQ reagents (114 and 115 for MAP 1018 grown in iron-replete and iron-limiting medium respectively; 116 and 117 for MAP 7565 grown in iron-replete and iron-limiting medium respectively) at lysine and arginine amino terminal groups. The labeled peptides were pooled, dried and re-suspended in 0.2% formic acid. The re-suspended peptides were passed through Oasis^® ^MCX 3CC (60 mg) extraction cartridges per manufacturer recommendations (Waters Corporation, Milford, MA) for desalting prior to strong cation exchange (SCX) fractionation.

Eluted peptides were dried and dissolved in SCX buffer A (20% v/v ACN and 5 mM KH_2_PO_4 _pH 3.2, with phosphoric acid) and fractionated using a polysulfoethyl A column (150 mm length × 1.0 mm ID, 5 μm particles, 300 Å pore size) (PolyLC Inc., Columbia, MD) on a magic 2002 HPLC system (Michrom BioResources, Inc., Auburn, CA). Peptides were eluted by running a 0-20% buffer B gradient for greater than 55 min. and 20%-100% buffer B (20% v/v ACN, 5 mM KH_2_PO_4 _pH 3.2, 500 mM KCL) for 20 min. at a column flow rate of 50 μl/min. Several fractions were collected at frequent intervals and seven fractions that showed mAU280 > 2 were analyzed by LC-MS/MS as previously described [28].

Fractions were reconstituted in reversed-phase load buffer (10 mM phosphate buffer) and analyzed in a 4800 MALDI TOF/TOF instrument (AB Sciex, Foster City, CA). Protein pilot Software™ 3.0.1 (AB Sciex, Foster city, CA) which utilizes the paragon™ scoring algorithm [29] was used to identify and quantify the relative abundance of the labeled peptides. Relative abundance of proteins (iron-replete v/s iron-limitation) for each MAP strain was determined by comparing the reporter ion ratios (114/115 for C and 116/117 for S MAP). iTRAQ experiments were repeated on two independent experiments for each treatment of each strain. We searched against the MAP K-10, non redundant (nr) mycobacteria proteins and entire nr protein database deposited in the NCBI along with the contaminants to identify MAP specific peptides at a false discovery rate of 1%.

## Results

### Transcriptional profiling of MAP IdeR

We recently characterized MAP IdeR and computationally predicted that IdeR in the presence of iron regulates expression of 24 genes [4]. In the current study, we identified that 20 of the 24 previously predicted genes were differentially expressed in response to iron by MAP microarrays. Mycobactin synthesis, transport and fatty acid biosynthesis genes were repressed in the presence of iron by both cattle and sheep MAP strains (Additional file 1, Table S2). However iron storage and oxidoreductase genes were upregulated in the presence of iron only in C MAP (Additional file 1, Figure S4).

We first confirmed if these differences are due to regulation via IdeR. IdeR is essential in MAP and attempts to delete this gene failed [26]. We complemented *M. smegmatis*Δ*ideR *(SM3) with C or S strain *ideR *and compared regulational differences in the presence or absence of iron. Genes that showed a log_2 _fold change of 1.0 in SM3 or SM3 complemented with empty plasmid (negative controls) in the presence or absence of iron while having a fold change >± 1.5 in the complemented strains (test) and plasmid carrying *M. smegmatis ideR *and *mc*^*2*^*155 *(wild type) (positive control) were considered as being regulated by MAP IdeR. Fourteen of the 20 genes were regulated by IdeRs of both MAP strains in *M. smegmatis. *Furthermore, our results suggested that sIdeR functions by primarily repressing genes in the presence of iron whereas cIdeR functions both by repressing mycobactin synthesis and de-repressing iron storage genes in the presence of iron (Additional file 1, Table S3). These were further validated by realtime RT-PCR in both *M. smegmatis *transformants carrying MAP *ideRs *and MAP genetic background. The data is presented only for MAP (Additional file 1, Table S4).

**Figure 1:**
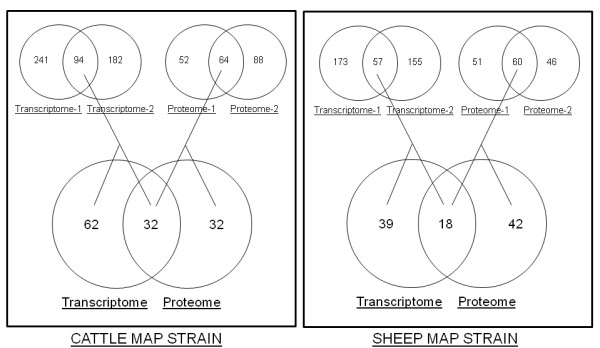
**Transcriptome and proteome comparisons: **Venn diagram showing the comparison of transcripts and proteins that were differentially expressed at a fold change of 1.5 or greater in cattle or sheep MAP strains in response to iron. One third of the genes differentially expressed in response to iron were represented in both the transcriptome and the proteome.

We next compared the transcriptome and proteomes of C and S MAP strains under iron-replete and iron-limiting conditions. Transcriptome analysis under two independent observations identified that 94 and 57 genes were consistently expressed by the C and S MAP strain respectively. Similarly, proteome data revealed a consistent expression of 64 and 60 proteins by the cattle and sheep MAP strains respectively. A comparison of these consistently detected transcripts and proteins revealed that, in the presence of iron, one third of the differentially regulated genes (P < 0.05) were represented both in the respective transcriptome and the proteomes of the two strains (Figure 1).

### Transcript profiles under iron-limiting conditions

Under iron-limiting conditions both the MAP strains showed increased transcription of genes belonging to mycobactin synthesis and esx-3, an essential secretory system of mycobactin biosynthesis (Additional file 1, Tables S2 - S5) [30].

C MAP showed increased transcription of genes belonging to ABC type transporter proteins, *suf *operon involved in Fe-S cluster assembly proteins (MAP1187-MAP1192), fatty acid biosynthesis operon (MAP3188-MAP3190) and a pyruvate dehydrogenase operon (MAP2307c-MAP2309c) (Table 1 and Additional file 1, Table S5) suggesting that the transcriptional machinery is used to mobilize iron to maintain intracellular homeostasis. CMAP also upregulated expression of an enhanced intracellular survival gene (*eis*) (MAP2325), which was described as "deletion 3" in sheep strains of MAP [16].

**Table 1:** Transcript and protein expression in cattle MAP under iron-limiting (LI) conditions

	MAP ORF ID	Predicted function	^**a**^**Fold change**	
			**Protein**	**Transcript**
			
Metabolism				
	MAP1587c	alpha amylase	2.03 ± 0.2	2.87 ± 0.7
	MAP1554c	FadE33_2 (acyl-coA synthase)	1.79 ± 0.5	1.88 ± 0.8
	MAP2307c	pdhC alpha-keto acid dehydrogenase	1.68 ± 0.3	2.52 ± 0.4
	MAP3189	FadE23 (acyl-CoA dehydrogenase)	2.41 ± 0.2	3.51 ± 1.0
	MAP3694c	FadE5 (acyl-CoA dehydrogenase)	1.87 ± 0.8	3.15 ± 0.2
Cellular processes				
	MAP3701c	heat shock protein	2.18 ± 0.6	2.48 ± 0.3
	MAP1188	FeS assembly protein SufD	2.23 ± 1.0	2.73 ± 0.2
	MAP1189	FeS assembly ATPase SufC	1.78 ± 0.5	2.03 ± 0.1
	MAP4059	heat shock protein HtpX	1.48 ± 0.1	1.66 ± 0.5
Poorly characterized pathways				
	MAP1012c	patatin-like phospholipase	1.67 ± 0.3	1.56 ± 0.3
	MAP1944c	iron suphur cluster biosynthesis	1.56 ± 0.9	1.66 ± 0.2
	MAP2482	Glyoxalase/Bleomycin resistance	1.84 ± 0.3	2.19 ± 0.8
	MAP3838c	RES domain containing protein	1.50 ± 0.7	2.40 ± 0.2

^a^MAP oligoarray was used to measure gene expression whereas iTRAQ was used to quantitate protein expression in the cultures of cattle MAP strain grown in iron-replete (**HI**) or iron-limiting (**LI**) medium. Fold change for each target was calculated and represented as a log_2 _ratio of LI/HI. Shown are the MAP genes that demonstrated the presence of 1.5 times or more of transcripts and proteins in LI compared to HI. Genes are annotated based on the motif searches in KEGG database.

In contrast, the sheep strain of MAP in addition to upregulation of putative iron uptake and transport genes also expressed those belonging to heat shock proteins, molecular chaperones, and a VapBC family of toxin-antitoxin operon (MAP2027c, MAP2028c) suggesting that iron deprivation might lead to a stringency response (Table 2 and Additional file 1, Table S6).

**Table 2:** Transcript and protein expression in sheep MAP under iron-limiting (LI) conditions

	MAP ORF ID	Predicted function	^**a**^**Fold change**	
			**Protein**	**Transcript**
			
Metabolism				
	MAP3564	methyltransferase	1.54 ± 0.1	1.58 ± 0.6
	MAP1942c	CbhK, ribokinase	1.74 ± 0.3	2.05 ± 1.0
	MAP2286c	thioredoxin domain containing protein	1.82 ± 0.1	2.04 ± 0.3
	MAP1997	acyl carrier protein	1.90 ± 0.5	1.68 ± 0.5
Cellular processes				
	MAP4340	TrxC, thioredoxin	1.50 ± 0.4	2.29 ± 0.3
	MAP3840	DnaK molecular chaperone	1.63 ± 0.6	3.52 ± 0.5
Information storage and processing				
	MAP4142	FusA, elongation factor G	1.52 ± 0.2	2.58 ± 0.7
	MAP4268c	transcriptional regulatory protein	1.52 ± 0.3	1.50 ± 0.1
	MAP4233	DNA-directed RNA polymerase alpha subunit	1.56 ± 0.1	1.83 ± 0.3
	MAP3024c	DNA binding protein, HU	1.60 ± 0.6	1.81 ± 0.5
	MAP4184	30S ribosomal protein S5	1.75 ± 0.1	1.55 ± 0.3
	MAP3389c	response regulator	1.94 ± 0.3	1.59 ± 0.2
	MAP4111	transcription antitermination protein, NusG	1.98 ± 0.3	1.82 ± 0.5
	MAP4143	elongation factor Tu	2.08 ± 0.4	2.16 ± 0.1
Poorly characterized pathways				
	MAP2844	conserved alanine and arginine rich protein	1.54 ± 0.2	2.27 ± 0.5
	MAP3433	initiation of DNA replication	1.63 ± 0.1	1.91 ± 0.2
	MAP0126	transcriptional regulator like protein	1.75 ± 0.6	1.50 ± 0.2
	MAP1065	pyridox oxidase	1.83 ± 1.0	1.52 ± 0.5

^a^MAP oligoarray was used to measure gene expression whereas iTRAQ was used to quantitate protein expression in the cultures of sheep MAP strain grown in iron-replete (**HI**) or iron-limiting (**LI**) medium. Fold change for each target was calculated and represented as a log_2 _ratio of LI/HI. Shown are the MAP genes that demonstrated the presence of 1.5 times or more of transcripts and proteins in LI compared to HI. Genes are annotated based on the motif searches in KEGG database.

### Transcript profiles under iron-replete conditions

There is increased protein synthesis and turnover in response to iron in *M. tuberculosis *(MTB) [31]. Similarly, the C strain upregulated as many as 25 rRNA genes, lipid metabolism, and several virulence-associated genes such as *fbpA *(MAP0216) of antigen85 complex, soluble secreted antigen (MAP2942c), and oxidoreductase (MAP1084c) (Tables 3 and Additional file 1, Table S7). There was also an upregulation of MAP3296c, a *whiB *ortholog of *M. tuberculosis *that plays a role in antibiotic resistance and maintains intracellular redox homeostasis [32]. Further, esx-5 operon which is present only in pathogenic mycobacteria and plays a role in cell-cell migration of mycobacteria was upregulated [33]. A hypothetical protein (MAP0860c) upregulated in the presence of iron in the cattle strain of MAP has been described as a part of MAP-specific large sequence polymorphism (LSP4) [22].

**Table 3:** Transcript and protein expression in cattle MAP under iron-replete (HI) conditions

	MAP ORF ID	Predicted function	^**a **^**Fold change**	
			**Protein**	**Transcript**
			
Metabolism				
	MAP0150c	FadE25_2 (acyl-coA dehydrogenase)	1.72 ± 0.1	1.88 ± 0.2
	MAP0789	acetyl-CoA acetyltransferase	1.73 ± 0.3	1.56 ± 0.1
	MAP1846c	ATP phosphoribosyltransferase	1.69 ± 0.2	3.68 ± 0.3
	MAP2332c	Fas (fatty acid synthase)	1.61 ± 0.5	2.28 ± 0.4
	MAP3404	AccA3 (acetyl-/propionyl-coenzyme A)	1.45 ± 0.1	2.18 ± 0.2
	MAP3698c	succinate dehydrogenase	1.89 ± 0.3	4.57 ± 0.5
Cellular processes				
	MAP1339	iron regulated conserved protein	1.62 ± 0.2	0.78 ± 0.3
	MAP1653	thiol peroxidase	1.79 ± 0.5	2.29 ± 0.2
Information storage and processing				
	MAP2907c	translation initiation factor IF-2	1.57 ± 0.2	1.89 ± 0.2
	MAP2945c	ribosome releasing factor	1.66 ± 0.3	2.11 ± 0.5
	MAP4113	50S ribosomal protein L1	1.61 ± 0.1	1.57 ± 0.2
	MAP4125	rplJ 50S ribosomal protein L10	1.52 ± 0.1	1.66 ± 0.5
	MAP4142	fusA elongation factor G	2.13 ± 0.4	3.05 ± 0.3
	MAP4160	rpsJ 30S ribosomal protein S10	1.68 ± 0.3	2.87 ± 0.4
	MAP4181	rpsH 30S ribosomal protein S8	1.79 ± 0.5	2.42 ± 0.1
	MAP4233	rpoA DNA-directed RNA polymerase	1.56 ± 0.1	1.65 ± 0.4
Poorly characterized pathways				
	MAP0216	FbpA antigen 85-A	1.87 ± 0.2	2.16 ± 0.3
	MAP1122	mycobacterial integration host factor	1.73 ± 0.3	2.00 ± 0.5

^a ^MAP oligoarray was used to measure gene expression whereas iTRAQ was used to quantitate protein expression in the cultures of cattle MAP strain grown in iron-replete (HI) or iron-limiting (LI) medium. Fold change for each target was calculated and represented as a log_2 _ratio of HI/LI. Shown are the MAP genes that demonstrated the presence of 1.5 times or more of transcripts and proteins in HI compared to LI. Genes are annotated based on the motif searches in KEGG database.

In contrast, we did not document any upregulation (at a log_2 _fold change of 1.5) in the S MAP under iron-replete conditions. The directionality of transcripts as identified by microarrays under iron-replete conditions by S MAP strain was confirmed by real time RT-PCR (Additional file 1, Table S4).

### Proteome

The following criteria were used for protein identification in each treatment - (1) peptides identified by mass spectrometry were searched against the non-redundant (nr) protein database deposited in NCBI); and (2) MAP specific peptides reported with >95% confidence were used to quantify the relative abundance (iron-replete v/s iron-limitation) of each protein. A peptide with no hits on the MAP genome but with identities with other mycobacterial proteins was considered as unannotated MAP protein.

### Protein expression under iron-limiting conditions

Consistent with the transcription profile, the C strain of MAP upregulated proteins belonging to SUF operon involved in Fe-S cluster assembly, fatty acid metabolism and a pyruvate dehydrogenase (MAP2307c). Transporter proteins, two component systems, and cell division associated proteins (MAP1906c, MAP0448 and MAP2997c) were also upregulated by the C strain (Table 1 and Additional file 1, Table S8). The sheep strain also upregulated transporter proteins, fatty acid biosynthesis, DNA replication protein (MAP3433), and stress response proteins (MAP3831c, MAP2764) (Table 2, Additional file 1, Table S9 and Figure S3).

**Figure 2:**
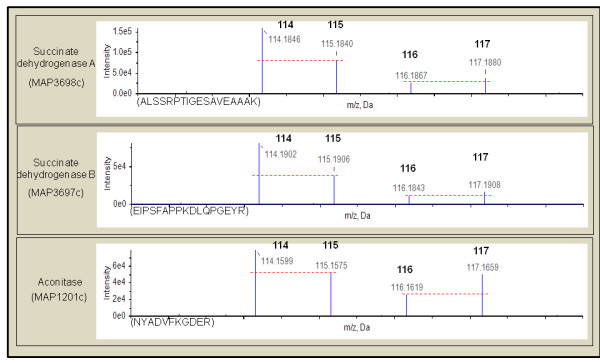
**Repression of non-essential iron using proteins under iron-limiting conditions by cattle MAP strain: **Reporter ion regions (114 - 117 *m/z*) of peptide tandem mass spectrum from iTRAQ labeled peptides from MAP3698c, MAP3697c and MAP1201c are shown. Quantitation of peptides and inferred proteins are made from relative peak areas of reporter ions. Peptides obtained from cattle MAP cultures grown in iron-replete and iron-limiting medium were labeled with 114 and 115 reporter ions, respectively.. Peptides obtained from sheep MAP cultures grown in iron-replete and iron-limiting medium were labeled with 116 and 117 reporter ions, respectively. The peptide sequences and shown in the parenthesis and the red dashed line illustrates the reporter ion relative peak intensities. Cattle strain of MAP shows an iron sparing response by downregulating expression of iron using proteins.

The iron-sparing response to iron starvation occurs when non-essential iron utilization proteins such as aconitase and succinate dehydrogenases are repressed and intracellular iron is used to maintain essential cellular functions [34,35]. Interestingly, during iron limitation, the cattle strain but not sheep MAP downregulated expression of aconitase (MAP1201c) and succinate dehydrogenases (MAP3697c, MAP3698c) (Figure 2). Repression of aconitase in response to iron starvation is post-transcriptionally mediated via small RNAs [36]. Consistent with this finding, our results reveal an upregulation of aconitase transcripts (both by microarray and Q-RT PCR) with a concomitant downregulation at the protein level in the C MAP alone under iron-limiting conditions.

### Protein expression under iron-replete conditions

**Figure 3:**
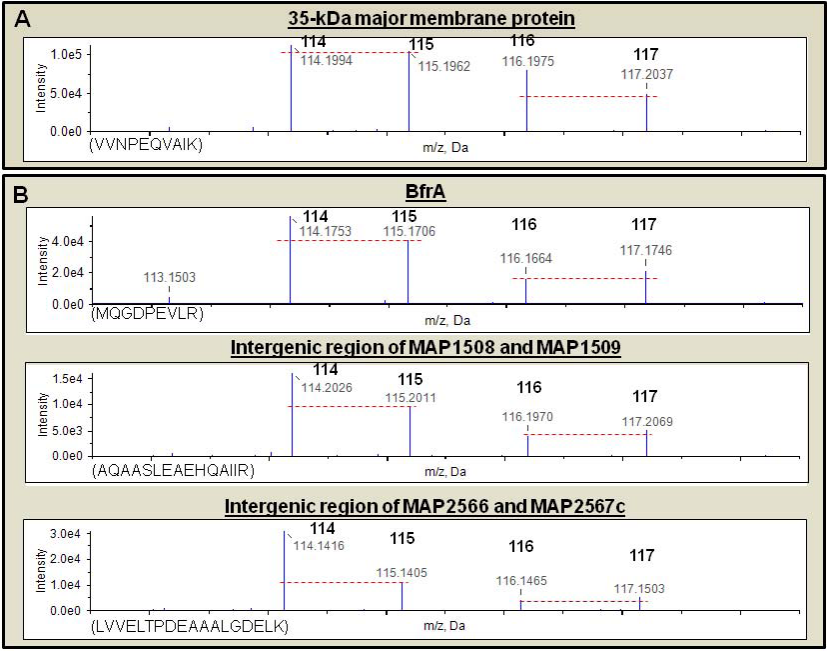
**Peptide quantitation of proteins expressed by C and S MAP strains under iron-replete conditions: **Reporter ion regions (114 - 117 *m/z*) of peptide tandem mass spectrum from iTRAQ labeled peptides from the (A) 35-kDa major membrane protein (MAP2121c) and (B) BfrA, and the intergenic regions of MAP1508-1509 and MAP2566-2567c. Quantitation of peptides and inferred proteins are made from relative peak areas of reporter ions. Several unique peptides (>95% confidence) were mapped to each protein. However, only one representative peptide is shown for each protein. Peptides obtained from cattle MAP cultures grown in iron-replete and iron-limiting medium were labeled with 114 and 115 reporter ions, respectively. Peptides obtained from sheep MAP cultures grown in iron-replete and iron-limiting medium were labeled with 116 and 117 reporter ions, respectively. The peptide sequences and shown in the parenthesis and the red dashed line illustrates the reporter ion relative peak intensities. MAP2121c alone was upregulated in the sheep MAP strain under iron-replete conditions.

The sheep strain upregulated as many as 13 unique peptides (>95% confidence) that were mapped to MAP2121c. A representative peptide map is shown in Figure 3A. Interestingly, none of these were differentially regulated in response to iron by C strain of MAP. MAP2121c was originally described as 35-kDa antigen and is an immune-dominant protein involved in MAP entry into bovine epithelial cells [37,38]. Although statistically not significant, further microarray analysis revealed a two-fold increase of MAP2121c in both cattle and sheep strains under iron-replete conditions (data not shown) suggesting a possible post transcriptional repression of MAP2121c by the cattle strain of MAP.

**Figure 4:**
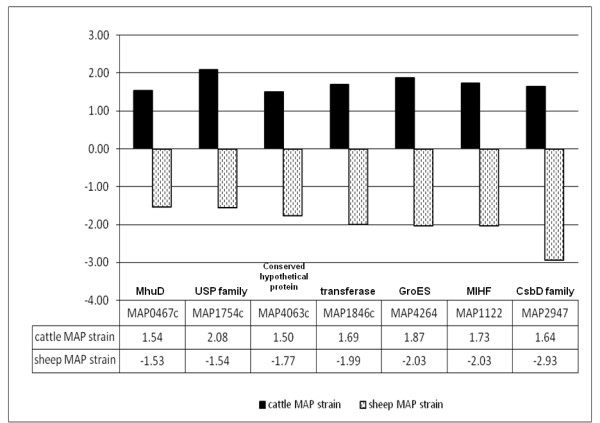
**Proteins expressed by type II MAP under iron-replete conditions: **Proteins upregulated in cattle MAP strain whereas downregulated in sheep strain in the presence of iron. Fold change for each target is calculated and represented as a ratio of iron-replete/iron-limitation. A negative fold change represents repression and a positive fold change indicates de-repression of that particular target gene in the presence of iron. MhuD = mycobacterial heme utilization, degrader; USP = universal stress protein; CHP = conserved hypothetical protein; MIHF = mycobacterial integration host factor; CsbD = general stress response protein

As expected, transcripts identified as upregulated under iron-replete conditions in C MAP strain were also upregulated in the proteome (Table 3, Additional file 1, Table S10). There was increased expression of five ribosomal proteins and a ribosome releasing factor (MAP2945c) by cattle MAP under iron-replete conditions. As previously reported, BfrA was upregulated in cattle MAP (Figure 3B). Antigen 85A and MAP0467c (mycobacterial heme, utilization and degrader) were also upregulated. However, MAP0467c and other stress response proteins were downregulated in the S MAP strain (Figure 4).

### Identification of unannotated MAP proteins

We identified two unique peptides (**SSHTPDSPGQQPPKPTPAGK **and **TPAPAKEPAIGFTR**) that originated from the unannotated MAP gene located between MAP0270 (fadE36) and MAP0271 (ABC type transporter). We also identified two peptides (**DAVELPFLHK **and **EYALRPPK**) that did not map to any of the annotated MAP proteins but to the amino acid sequence of MAV_2400. Further examination of the MAP genome revealed that the peptides map to the reversed aminoacid sequence of MAP1839. These two unique proteins were not differentially regulated in response to iron. However, two more unique peptides that were translated from other unannotated MAP genes were upregulated (>1.5 fold) under iron-replete conditions in C MAP strain (Figure 3B).

## Discussion

Johne's disease is a major animal health problem of ruminant species worldwide and imposes significant economic losses to the industry. Our ability to culture the causative agent--*Mycobacterium avium *subsp. *paratuberculosis *(MAP)--and therefore its rapid diagnosis and our understanding of its virulence is limited. MAP is difficult to culture because of its unusually strict iron requirements. For optimal growth in laboratory media, MAP requires a siderophore (mycobactin) supplementation that makes MAP fastidious [39]., often requiring eight to sixteen weeks to produce colonies in culture - a major hurdle in the diagnosis and therefore implementation of optimal control measures. Understanding iron regulatory networks in the pathogen invitro is therefore of great importance.

### A tale of two strain types of MAP - A case to study iron regulation

Several microbiological and genotyping studies and clinical observations suggest that Johne's in certain hosts such as sheep, goats, deer, and bison is caused by a distinct set of strains that show a relatively high degree of host preference [18,40]. At least two microbiologically distinct types of MAP have been recognized. A less readily cultivable type is the common, but not invariable, cause of paratuberculosis in sheep (S MAP) [39,41,42], while another readily cultivable type is the most common cause of the disease in cattle (C MAP). Cell infection studies have also revealed distinctive host response phenotypes between cattle and sheep MAP strains - the former elicit primarily a pro-inflammatory response while latter strains suppress inflammation and upregulate anti-apoptotic pathways [24,25]. In addition, since *MAP *genome sequence was published in 2005, very little research has focused on iron physiology and its contribution to metabolic networks of this fastidious organism.

Based on these classical microbiologic, genotypic, and clinical observations, we addressed the hypothesis that the iron dependent gene regulation is different between cattle and sheep MAP strains using a systems approach.

### Iron-sparing response to iron-limitation is unique to cattle MAP strain

Iron is a critical component of several metabolic enzymes [43]. Most bacteria respond to iron starvation with a unique regulatory mechanism called the iron-sparing response [35]. Iron-sparing is a physiological phenomenon used by cells to increase the intracellular iron pool by post-transcriptionally repressing the synthesis of non-essential iron using proteins and sparing iron for essential cellular functions [44]. Therefore, the paradigm is to transcriptionally upregulate all iron uptake systems while repressing non-essential enzymes via post-transcriptional regulatory mechanisms to survive iron-limiting conditions. Both MAP strains upregulated genes involved in siderophore biosynthesis (*mbt*), ability to acquire iron from synthesized siderophores (*esx-3*), and to transport iron bound siderophores (*irtAB*) into the bacterium (Additional file 1, Table S2). Furthermore, cattle MAP strain under iron-limiting conditions upregulated transcription of aconitase (Additional file 1, Table S4) while downregulating its protein expression (Figure 2). It is likely that targets for post-transcriptional repression of these non-essential iron using proteins are mediated via small RNAs [34]. Studies to test this hypothesis in the two MAP strain types are underway.

### Differential metabolic responses of cattle and sheep MAP strains to iron-limitation

Under iron-limiting conditions most other bacteria including *M. tuberculosis *(MTB) upregulate SUF operon [26,45]. SUF synthesizes [Fe-S] clusters and transports them to iron-sulfur containing proteins involved in diverse cellular functions such as redox balance and gene regulation [46]. This is critical because unlike *E. coli*, MTB and MAP genomes encode for only one such system to synthesize all the [Fe-S] needed by the cell and free iron and sulfide atoms are toxic to cells [47]. Our data revealed that cattle strain, but not S strain upregulated SUF operon at the transcript and protein level under iron-limiting conditions (Table 1).

Cattle MAP strain upregulated pyruvate dehydrogenase operon involved in catabolism of propionate a key component of lipid biosynthesis under limiting iron conditions [48]. In contrast, sheep strain upregulated isoprenoid synthesis genes involved in cell wall biogenesis [49]. The sheep isolate also upregulated oxidoreductase and stress responses in its transcriptome and proteome during iron-limitation (Table 2). CarD and toxin-antitoxin systems primarily function during unfavorable conditions such as starvation or oxidative stress by arresting cell growth [50,51]. Sheep strain upregulated transcripts of toxin-antitoxin system involved in arresting cell growth, suggesting a trend toward stringency response (Additional file 1, Table S6). Taken together, our data suggests that cattle strain is able to efficiently modulate its metabolism during iron-limitation - probably a survival advantage.

MAP2325, a hypothetical protein deleted in the sheep strain was found to be upregulated under iron-limiting conditions by the C strain (Additional file 1, Table S5). This is interesting because an ortholog of MAP2325 in MTB called *enhanced intracellular survival *(*eis*) interacts with host T cells. Stimulation of recombinant Eis from MTB results in increased production of IL-10 and decreased production of TNF-α thus contributing to mycobacterial survival inside macrophages [52]. We have also demonstrated a similar result in bovine or human macrophages stimulated with diverse MAP strains. Cattle strains produced relatively more IL-10 and less TNF-α and persisted for longer periods of time inside macrophages [24,25].

There is increased protein synthesis and turn over in response to iron in MTB [31]. Similarly, we observed an increased expression of ribosomal proteins in the transcriptome and proteome in C MAP under iron-replete conditions. In striking contrast, iron-limitation induced a similar theme in sheep strain. Heme degradation is a significant physiological phenomenon where in pathogens recycle iron and gain a survival advantage inside the host [53]. Recently the crystal structure of Rv3592 of MTB was solved and demonstrated its ability as heme degrader [54]. We observed an upregulation of MAP0467c protein (ortholog of Rv3592) under iron-replete conditions in C MAP while it was downregulated in the sheep strain (Figure 4). Similar to our previous reports, iron storage protein, BfrA was upregulated only by C MAP under iron-repletion (Figure 3) [4]. Although the reasons for differential iron storage mechanisms in sheep compared to cattle strains of MAP are currently unknown, differential role of ferritins in bacterial pathogens is not uncommon [55].

## Conclusions

**Figure 5:**
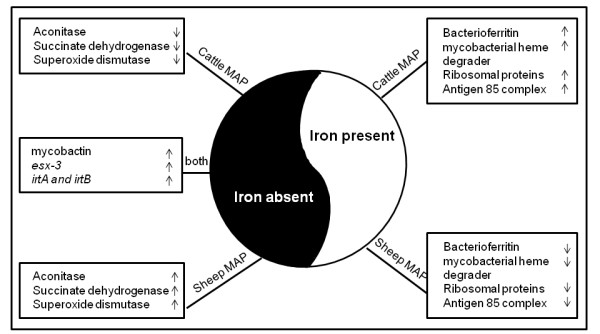
**Iron dependent metabolic programming in cattle and sheep MAP: **Under iron-replete conditions, there is upregulation of ribosomal proteins, bacterioferritin, mycobacterial heme, utilization and degrader proteins in cattle strain alone. Under iron limiting conditions, siderophore synthesis and transport genes are upregulated in both cattleI and sheep MAP strains. However, under iron limitation there is downregulation of aconitase, succinate dehydrogenases and superoxide dismutase in cattle MAP strain alone. This suggests an iron-sparing response exclusively in cattle but not sheep strain.

Our data revealed striking differences in metabolic pathways used by cattle and sheep strains of MAP to adapt to iron starvation (Figure 5). We have identified and characterized key iron dependent pathways of MAP. Since iron metabolism is critical for the invivo and invitro survival of the bacterium, our current studies are expected to improve our ability to provide better invitro culture methods for MAP and provide an understanding of iron regulation as a key virulence determinant of MAP.

## Authors' contributions

SS designed the study. HKJ participated in the experimental design with SS and performed most of the experiments. SK and AK helped in some experiments. JBP contributed to new reagents. BAW performed mass spectrometry. PJ and LAH helped in iTRAQ data analysis. HKJ and SS analyzed the data and wrote the manuscript. All authors read and approved the manuscript.

## Supplementary Material

Additional file 1 **Descriptive and pathway analysis of transcriptome and proteome data**. This file contains the experimental design, additional microarray, proteomic and Q-RT PCR data along with pathway analysis of iron stress response proteins of C and S MAP strains. Click here for file
